# Synthesis of a Hexameric Magnesium 4-pyridyl Complex with Cyclohexane-like Ring Structure via Reductive C-N Activation

**DOI:** 10.3390/molecules26237214

**Published:** 2021-11-28

**Authors:** Samuel R. Lawrence, Matthew de Vere-Tucker, Alexandra M. Z. Slawin, Andreas Stasch

**Affiliations:** EaStCHEM School of Chemistry, University of St Andrews, North Haugh, St Andrews KY16 9ST, UK; sl264@st-andrews.ac.uk (S.R.L.); mdvt@st-andrews.ac.uk (M.d.V.-T.); amzs@st-andrews.ac.uk (A.M.Z.S.)

**Keywords:** bond activation, low oxidation state complexes, magnesium, metallacycles, ring system, X-ray crystallography

## Abstract

The reaction of [{(^Ar^nacnac)Mg}_2_] (^Ar^nacnac = HC{MeC(NAr)}_2_, Ar = 2,6-diisopropylphenyl, Dip, or 2,6-diethylphenyl, Dep) with 4-dimethylaminopyridine (DMAP) at elevated temperatures afforded the hexameric magnesium 4-pyridyl complex [{(^Ar^nacnac)Mg(4-C_5_H_4_N)}_6_] via reductive cleavage of the DMAP C-N bond. The title compound contains a large *s*-block organometallic cyclohexane-like ring structure comprising tetrahedral (^Ar^nacnac)Mg nodes and linked by linear 4-pyridyl bridging ligands, and the structure is compared with other ring systems. [(^Dip^nacnac)Mg(DMAP)(NMe_2_)] was structurally characterised as a by-product.

## 1. Introduction

**Figure 1:**
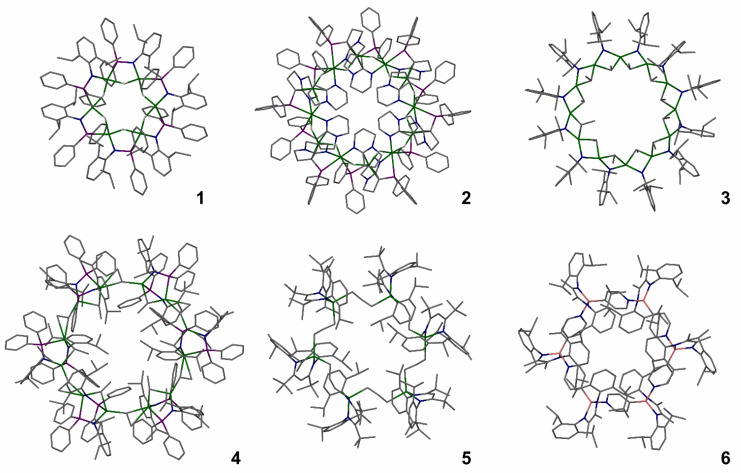
Overall molecular structures of complexes **1**–**6**. Hydrogen atoms, except MgH units, omitted for clarity. Colour code: P: purple, Al: pink, Mg: green, N: blue, C: grey, hydridic H: light grey.

The study of self-assembly in metallacycles via coordination chemistry is dominated by transition metal complexes [1,2]. In these, the coordination geometry around transition metal centres is relatively strictly governed by their number of *d*-electrons and ligand field effects. Thus, the structure of metallacycles and coordination polymers can often be guided by the shape of connecting ligands and the position of donor functionalities together with suitable transition metal ions. In contrast, metal–ligand interactions in *s*-block metal complexes are predominantly electrostatic in nature [3]. This can lead to reversible metal–ligand coordination bonds with flexible coordination modes that are easy to distort from “ideal” geometry. Often, many complex species of similar energy are present in solution, that rapidly interconvert with low barriers, and are significantly influenced by ligand sterics, donor solvents and other factors. Thus, for a range of *s*-block metal complexes, equilibria between various oligomers, such as ring systems, can make it difficult to predict the range of possible structures. In addition to solution state spectroscopic studies, single crystal X-ray crystallography has been crucial in elucidating often surprising molecular structures, that were sometimes obtained serendipitously, and has allowed linking to solution state species. For coordination complexes with electropositive divalent metal centres, e.g., Mg^2+^ in organomagnesium or amidomagnesium compounds, ring systems have often been discovered when two types of anionic, often bridging ligands with unequal sizes are present, that induce a curvature and can lead to ring formation [4]. Relevant Mg complexes, **1**–**5**, with hexameric or dodecameric ring structures are shown in Figure 1. These examples show that smaller bridging ligands (hydride, ethyl, allyl) are located on the inside of the ring, whereas the more sterically demanding and solubilising ligands, most of them containing 2,6-diisopropylphenyl (Dip) substituents, are predominantly located towards the outside of the ring. The examples all contain sterically demanding monoanionic bridging (**1** [5], **2** [4], **3** [6], **4** [5]) or chelating (**5** [7]) ligands, plus small bridging ligands such as hydrides (**1** and **2**), ethyl groups (**3** and **4**) or allyl groups (**5**). Placing the larger ligands with solubilising groups on the outside of the ring will contribute to the solubility of the molecules, and thus may aid the formation of a preferred ring size through self-assembly. The general bond lability, and typical reversibility, of ionic metal–ligand interactions is a process for interconverting different oligomers to a thermodynamically preferred size and/or to those favoured by crystallisation from reaction mixtures under certain conditions. Very recently, and related to the work in here, a blue aluminium complex [{(MeCNDip)_2_Al(4-C_5_H_4_N)}_6_] **6** (see Figure 1) bearing a sterically demanding diazabutadiene-diide ligand, aluminium(III) centres and 4-pyridyl units, was prepared by pyridine CH activation with hydrogen elimination of a respective aluminium(II) complex with pyridine at elevated temperatures [8].

**Figure 2:**
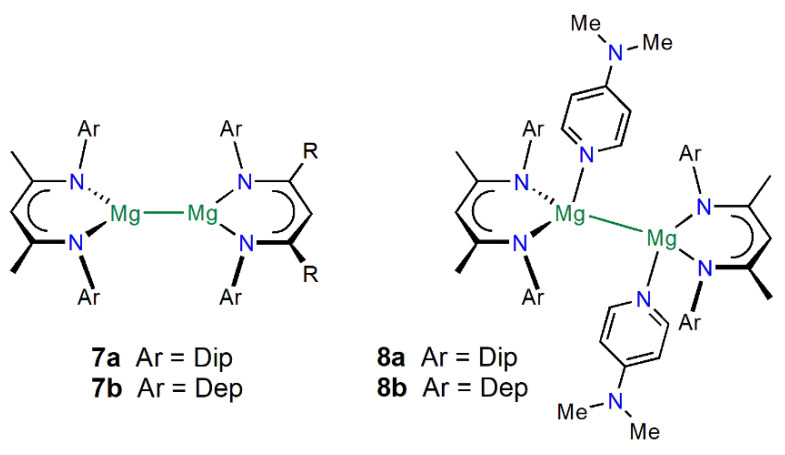
Dimagnesium(I) complexes **7** and **8**. Dip = 2,6-diisopropylphenyl, Dep = 2,6-diethylphenyl.

In magnesium complex chemistry [9], dimagnesium(I) compounds of general type LMgMgL [10,11,12], where L is a monoanionic ligand such as in the β-diketiminate examples **7**, see Figure 2, are highly reducing and highly reactive reagents that can also serve as entry points to heteroleptic magnesium complexes with unusual anionic fragments. The magnesium atoms in LMgMgL can be coordinated by additional donor molecules, for example with substituted pyridines or cyclic ethers [13,14,15,16]. This can lead to complexes with significantly elongated Mg–Mg bonds, e.g., in the bis-DMAP adduct [{(^Ar^nacnac)Mg(DMAP)}_2_] **8**, where ^Ar^nacnac = HC{MeC(NAr)}_2_, with Ar = Dip (**8a**) [13] or Dep (2,6-diethylphenyl) (**8b**) [16], and DMAP = 4-dimethylaminopyridine, see Figure 2, with an elongation by ca. 12% in **8a** (Mg–Mg: 3.1962(14) Å) from ca. 2.85 Å in **7a** [13,17]. Coordination of ligands can be reversible, e.g., for cyclic ethers such as THF and dioxane, and the reactivity at the Mg centres of LMgMgL compounds is typically suppressed when fully coordinated. Mono-adducts of LMgMgL complexes, e.g., with DMAP or small *N*-heterocyclic carbene species, or partial donor addition, however, can induce reactivity not observed for the uncoordinated species. This has, for example, been demonstrated in the reductive coupling of CO [15,16].

## 2. Results and Discussion

### 2.1. Synthesis

**Scheme 1:**
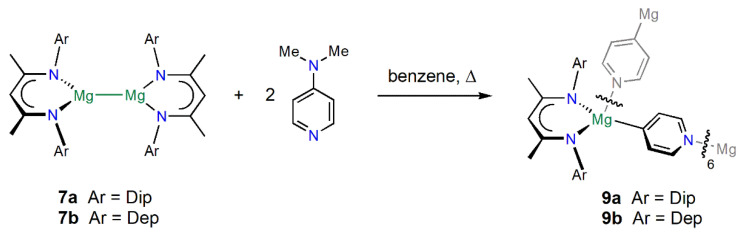
Synthesis of complexes **9**.

**Figure 3:**
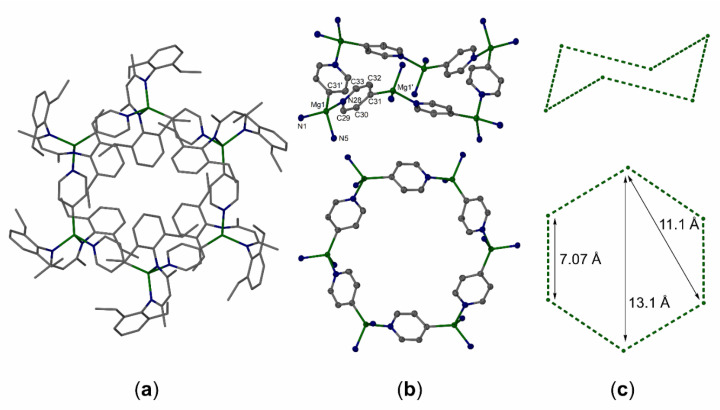
Molecular structure of [{(^Dep^nacnac)Mg(4-C_5_H_4_N)}_6_]·C_6_H_6_, **9b**·C_6_H_6_. Solvent molecule, hydrogen atoms and minor part of a disordered ethyl group omitted. Colour code: Mg: green, N: blue, C: grey. (**a**) Stick diagram of overall structure; (**b**) cut-back model as thermal ellipsoids (30%) in two views with atom labelling of asymmetric unit and nearest atoms; (**c**) only magnesium positions in two views and selected distances. Selected bond lengths (Å) and angles (°): Mg1–N1 2.0410(12), Mg1–N5 2.0505(12), Mg1–N28 2.1138(12), Mg1′–C31 2.1462(14), N28–C29 1.3437(18), C29–C30 1.379(2), C30–C31 1.4004(19), C31–C32 1.405(2), C32–C33 1.380(2), N28–C33 1.3411(18); N1–Mg1–N5 93.09(5), N28–Mg1–C31′ 106.84(5).

The reaction of [{(^Ar^nacnac)Mg}_2_] **7**, Ar = Dip (**7a**) [17] or Dep (**7b**) [18] with DMAP initially led to brown-red solutions of the adduct complexes **8**. [13,16] Upon heating to 100 °C, the colour of the mixtures changed to purple, and after several hours at this temperature, the hexameric magnesium 4-pyridyl complexes [{(^Ar^nacnac)Mg(4-C_5_H_4_N)}_6_], Ar = Dip (**9a**), Dep (**9b**) were reproducibly afforded (see Scheme 1). The compounds were obtained as colourless crystals when performed in a non-stirred reaction mixture such as an NMR tube, or as a crystalline precipitate from a stirred reaction in low to moderate isolated yields. Complex **9** shows a large cyclohexane-like ring structure (Figure 3) that is described in the next section in more detail. Once crystallised, the complexes did not redissolve in hot deuterated benzene, hot deuterated THF or dichloromethane, and thus no meaningful NMR spectroscopic data could be obtained. For comparison, NMR spectra of aluminium complex **6** have been recorded in deuterated THF. [8] The bulk purity of compound **9** is supported by elemental analysis. In addition, hydrolysis of **9b** with D_2_O in aromatic solvents afforded only 4-deuteropyridine and the deuterated proligand ^Dep^nacnacD in a 1:1 ratio, and no DMAP or other organic by-products according to ^1^H and ^2^H-NMR spectroscopy, plus a precipitate presumed to be Mg(OD)_2_·D_2_O. Similarly, the attempt to dissolve **9b** in deuterated dimethyl sulfoxide afforded a 1:1 mixture of 4-deuteropyridine and one β-diketiminate complex, by implication [(^Dep^nacnac)Mg{OS(CD_3_)CD_2_}], and no ^Dep^nacnacD, as judged by ^1^H and ^2^H-NMR spectroscopy.

**Figure 4:**
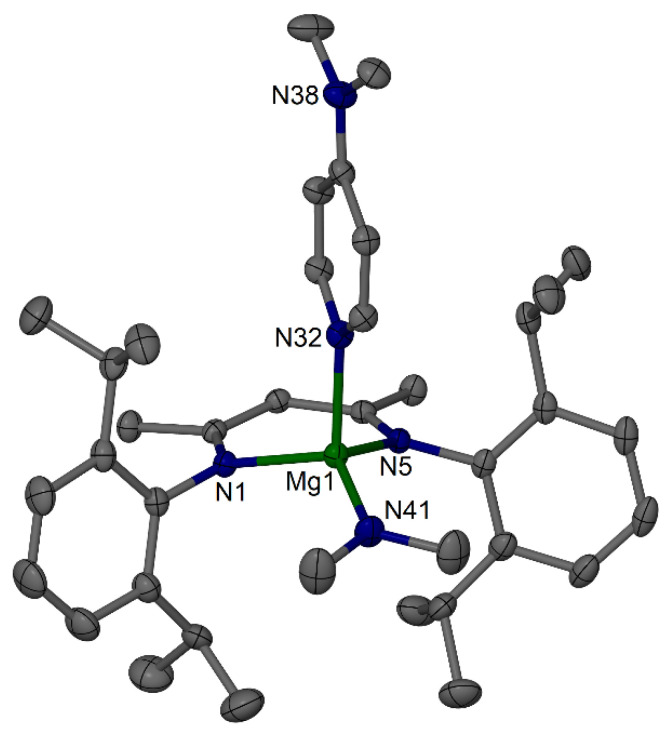
Molecular structure of [(^Dip^nacnac)Mg(DMAP)(NMe_2_)]·0.5 C_5_H_12_, **10**·0.5 C_5_H_12_, 30% thermal ellipsoids. Solvent molecule, hydrogen atoms and minor parts of disordered isopropyl groups omitted. Selected bond lengths (Å) and angles (°): Mg1–N1 2.0738(11), Mg1–N5 2.0756(11), Mg1–N32 2.1248(12), Mg1–N41 1.9468(12); N1–Mg1–N5 91.83(4), N41–Mg1–N32 108.39(5).

Both radical and diamagnetic reaction pathways can be considered for the observed reactivity and the reductive cleavage of the C-N bond by the Mg^I^ centres. The deeply coloured reaction mixtures, which decolourise rapidly when exposed to air, the high reaction temperature, and the extremely long and thus weakened Mg–Mg bond in the intermediate [{(^Ar^nacnac)Mg(DMAP)}_2_] **8** would support both possibilities, but point to a radical mechanism. ^1^H-NMR spectroscopic studies of the supernatant solution, after product **9** precipitated, showed that a mixture of products remained, with overlapping NMR resonances from several β-diketiminate-containing complexes. Small quantities of complex **8** could be identified in these mixtures. Attempts were made to isolate by-products from these reactions and the new complex [(^Dip^nacnac)Mg(DMAP)(NMe_2_)] **10** was structurally characterised after crystallisation from *n*-pentane in a low yield, see Figure 4 (next section), and is an expected by-product from the DMAP C–N bond scission. For the formation of the related Al complex **6** derived from pyridine, a radical mechanism was proposed based on DFT studies that initiated with the homolytic cleavage of the Al–Al bond. [8] When we carried out analogous reactions of compounds **7** to **9** with pyridine instead of DMAP, we again observed highly coloured reaction mixtures at 100 °C (green to purple-blue) and no pure compounds could so far be isolated from these reactions. In situ NMR spectroscopic studies showed significant broadening and a decrease in intensities of resonances which could suggest the formation of radical species. No significant quantities of hydrogen were formed in these reactions (*c.f.* the synthesis of **6**). In transition metal chemistry, formation of the tetrameric β-diketiminate Fe 4-pyridyl species [{MeC{MeC(NXyl)}_2_Fe(4-C_5_H_4_N)}_4_], Xyl = 2,6-dimethylphenyl, from an iron(I) complex and DMAP, was shown to involve pyridyl radical anions and an analogous Fe complex to **10**, [MeC{MeC(NXyl)}_2_Fe(DMAP)(NMe_2_)], was also isolated. [19] Radical intermediates have previously been generated in β-diketiminate dimagnesium(I) chemistry, either as intermediates or from photochemically induced Mg–Mg bond cleavage [20,21]. Reductions in organic substrates with **7** can also form complexes with organic-based radicals, for example a structurally characterised purple-blue magnesium ketyl complex [22]. Supported by DFT studies, the activation of CO via mono-adducts of **7** is, however, proposed to proceed via a diamagnetic pathway [15,16]. Overall, it is likely that the mechanism involves the Mg-induced reduction in the DMAP ligand to a radical anion and concomitant loss of the Mg–Mg bond, followed by reductive C–N cleavage and ultimately Mg–C bond formation.

### 2.2. Molecular Structures

Complexes [{(^Ar^nacnac)Mg(4-C_5_H_4_N)}_6_] Ar = Dip (**9a**), Dep (**9b**), were characterised by single crystal X-ray diffraction, see Figure 3 for **9b**, and Figures S1 and S2 for **9a**. Both compounds show a similar overall arrangements of alternating (^Ar^nacnac)Mg units and 4-pyridyl groups that form a large, cyclohexane-like ring system in a chair conformation where the (^Ar^nacnac)Mg units serve as tetrahedral nodes and the 4-pyridyl groups act as linear linking units and coordinate via the pyridyl nitrogen to one Mg, and the 4-pyridyl carbon to another Mg centre (*μ*-κ*N*:κ*C*^4^). Several datasets from different crystals were obtained for each compound and the data quality, refinement parameters and overall ordering in the crystal were repeatedly significantly better for **9b** (Ar = Dep; best *R* value ca. 4.5%) compared with **9a** (Ar = Dip; best *R* value ca. 15%). This is likely in part related to the differences in the substituents in the 2 and 6 position of the flanking aryl groups on the diketiminate ligand. The increased steric demand of the isopropyl groups in **9a** near the connecting 4-pyridyl groups appears to distort from the ideal geometry which likely translates to poor overall ordering and crystal quality. The ethyl groups in these positions in **9b** are more open, flexible, and accommodating. The other major difference is that solvates of **9b** included one benzene molecule per full formula unit, whereas those of **9a** contained an estimated 10 or 16 benzene molecules. Another contributing factor for the overall crystal quality in **9b** over **9a** could be that compounds with Dep substituents are typically more soluble than those with Dip substituents, and thus crystal formation and growth could be slower and thus provide higher quality crystals. The isolated yields were also higher for **9b** than for **9a** and that could relate to more facile ring formation.

The molecular structures of [{(^Dep^nacnac)Mg(4-C_5_H_4_N)}_6_]·C_6_H_6_, **9b**·C_6_H_6_, are broadly similar and only the highest quality example will be described in more detail. **9b**·C_6_H_6_ crystallised in a trigonal crystal system with one sixth of the molecule in the asymmetric unit and a disordered benzene molecule in the centre of the ring. Each Mg centre is four-coordinate with a 4-pyridyl Mg-C interaction (Mg1–C31′ 2.1462(14) Å) to a pyridyl with approximate co-planar orientation relative to the diketiminate-Mg unit and a 4-pyridyl Mg-N interaction (Mg1–N28 2.1138(12)) to a pyridyl that is approximately perpendicular to the diketiminate-Mg unit. A similar overall arrangement of carbon versus nitrogen atom positions is also found in the molecular structure of Al complex **6**. [8] Exchanging the pyridyl carbon versus nitrogen atom positions in the structure of **9b** also leads to similar reasonable thermal ellipsoids and *U*_iso_ values for the atoms in question, but the final *R* value of the refinement increases by more than 1%. The bond lengths within the pyridyl unit further support the assignment of the nitrogen atom position with significantly shorter C-N bonds (1.342 Å mean) versus C-C bonds (1.403 Å mean). The pyridyl N-Mg-C angle in **9b** is 106.84(5)° and thus close to an ideal tetrahedral angle. The 4-pyridyl units do not provide a perfectly linear coordination motif between two Mg centres and show a Mg∙∙∙4-pyridyl-midpoint∙∙∙Mg angle of ca. 170°. In the related tetrameric iron complex [{MeC{MeC(NXyl)}_2_Fe(4-C_5_H_4_N)}_4_], the coordination geometry around the iron(II) centres is distorted towards trigonal pyramidal with smaller pyridyl-Fe-pyridyl angles of 102.0° (mean), and two alternating types of Fe∙∙∙4-pyridyl-midpoint∙∙∙Fe angles of 173.8° (mean) and 159.1° (mean) highlighting the distortion in this system [19]. In addition, transition metal-coordinated 4-pyridyl units have been employed in the formation of organometallic coordination polymers derived from bis(4-pyridyl)mercury [23,24].

The ring system with alternating tetrahedral (^Ar^nacnac)Mg nodes and linear 4-pyridyl ligands is thus perfectly suited to form a large cyclohexane-like molecule in a chair configuration where the tetrahedrally coordinated Mg centres represent the carbon atoms and the linear 4-pyridyl ligands represent the C-C single bonds. In that sense, the overall structure of complex **9** is similar to that of the hexameric β-diketiminate magnesium allyl complex **5** [7], although the bridging allyl groups in **5** are expected to be coordinatively highly flexible, whereas the 4-pyridyl units in **9** provide a rigid bridging unit. Mg ring systems **1**-**4** appear to form due to the different sizes of bridging ligands and other oligomers are likely present in solution. For example, hexameric ring system **1** is a rare aggregate and a tetrameric structure was predominantly obtained [5].

Complex [(^Dip^nacnac)Mg(DMAP)(NMe_2_)]·0.5 C_5_H_12_, **10**·0.5 C_5_H_12_, crystallised with a full molecule in the asymmetric unit, see Figure 4. The overall arrangement of the neutral DMAP unit, approximately perpendicular to the diketiminate-magnesium plane, and the approximately co-planar arrangement of the anionic dimethylamide unit is typical for these types of complexes, for example in β-diketiminate magnesium butyl complexes with pyridine donor ligands [25,26,27], and similar to the arrangement in **9**. The dative Mg-N(DMAP) distance in **10** (2.1248(12) Å) is almost unchanged compared with that of the Mg-N(4-pyridyl) fragment in **9**, which is further support of the latter as a dative bond. The terminal Mg-amide bond in **10** (1.9468(12) Å) is significantly shorter.

## 3. Conclusions

In summary, the synthesis of the large hexameric magnesium 4-pyridyl ring system [{(^Ar^nacnac)Mg(4-C_5_H_4_N)}_6_] **9** via the reductive C-N cleavage of DMAP using dimagnesium(I) complexes at elevated temperatures is reported. [(^Dip^nacnac)Mg(DMAP)(NMe_2_)] **10** was structurally characterised as a by-product. Complex **9** shows a large cyclohexane-like ring structure that forms due to the alternating arrangement of tetrahedral (^Ar^nacnac)Mg units connected via linear 4-pyridyl groups that act as a rigid bridging ligand. This forms a rare organometallic *s*-block metallacycle that was compared with examples from the *p*- and *d*-block. A minor modification of the 2,6-substituents (isopropyl for **9a**, ethyl for **9b**) on flanking aryl groups on the ligand repeatedly led to large differences in the crystal quality. This work highlights again the importance of single crystal X-ray diffraction for the elucidation of unusual molecular structures of *s*-block metal complexes.

## 4. Materials and Methods

### 4.1. Experimental Details

All manipulations were carried out using standard Schlenk line and glove box techniques under an atmosphere of high purity dinitrogen or argon. Benzene and *n*-pentane were dried and distilled from LiAlH_4_ under inert gas. ^1^H and ^2^H-NMR spectra were recorded on a Bruker Avance 400 or AVIII 500 spectrometer in dried deuterated solvents and were referenced to the residual ^1^H-NMR resonances, or in benzene and were locked and referenced to the residual D_2_O peak (^2^H-NMR spectra). Melting points were determined in sealed glass capillaries under argon and are uncorrected. Elemental analyses were performed by the Elemental Analysis Service at London Metropolitan University. The elemental analyses were affected by the highly air and moisture sensitive nature of the compounds. [{(^Dip^nacnac)Mg}_2_] **7a** [17] and [{(^Dep^nacnac)Mg}_2_] **7b** [18] were prepared according to literature procedures. DMAP was used as received from Fluorochem [Hadfield, UK]. DMSO-*d*_6_ was dried over activated molecular sieves, degassed, and stored under inert gas.

### 4.2. Synthesis of [{(^Dip^nacnac)Mg(4-C_5_H_4_N)}_6_] 9a and Formation of [(^Dip^nacnac)Mg(DMAP)(NMe_2_)] 10

A J.Young flask was charged with [{(^Dip^nacnac)Mg}_2_] **7a** (200 mg, 226 μmol, 1 equiv.) and DMAP (55.3 mg, 453 μmol, 2 equiv.), and benzene (10 mL) was added. The resultant brown-red solution was heated to 100 °C for 16 h with stirring and resulted in a deep purple solution and an off-white precipitate. This solid was isolated by filtration and dried in vacuo, to give [{(^Dip^nacnac)Mg(4-C_5_H_4_N)}_6_] **9a** as a highly insoluble powder. Yield: 52.7 mg (22%, based on all Mg atoms). The filtrate was dried in vacuo and extracted into *n*-pentane (*ca.* 5 mL) to give a deep purple solution. Storage of this solution at room temperature for seven days yielded a crop of a mixture of compounds (according to ^1^H-NMR spectroscopy) from which a yellow crystal of [(^Dip^nacnac)Mg(DMAP)(NMe_2_)]·0.5 C_5_H_12_, **10**·0.5 C_5_H_12_ was analysed by single crystal X-ray diffraction. Crystals of [{(^Dip^nacnac)Mg(4-C_5_H_4_N)}_6_]·x C_6_H_6_, **9a**·x C_6_H_6_, (x ≈ 10 or 16, see the X-ray section) were obtained directly from unstirred reaction mixtures. Data for **9a**: M.p.: 214–222 (decomposition to black solid); elemental analysis (C,H,N, combustion) for C_204_H_270_Mg_6_N_18_, found: C, 77.13; H, 8.45; N, 7.75; calc: C, 78.52; H, 8.72; N, 8.08.

### 4.3. Synthesis of [{(^Dep^nacnac)Mg(4-C_5_H_4_N)}_6_] 9b and Hydrolysis/Deuteronation

A J.Young flask was charged with [{(^Dep^nacnac)Mg}_2_] **7b** (200 mg, 259 μmol, 1 equiv.) and DMAP (63.3 mg, 518 μmol, 2 equiv.), and benzene (10 mL) was added. The resultant brown-red solution was heated to 100 °C for 16 h with stirring and resulted in a deep purple solution and an off-white precipitate. The solid was isolated by filtration and dried in vacuo, to give [{(^Dep^nacnac)Mg(4-C_5_H_4_N)}_6_] **9b**·as a highly insoluble powder. Yield: 85.0 mg (35%, based on all Mg atoms). Crystals of [{(^Dep^nacnac)Mg(4-C_5_H_4_N)}_6_]·C_6_H_6_, **9b**·C_6_H_6_, were obtained directly from unstirred reaction mixtures. Data for **9b**: M.p.: 199-207 (decomposition to black solid); elemental analysis (C,H,N, combustion) for C_186_H_228_Mg_6_N_18_, found: C, 77.30; H, 7.92; N, 8.37; calc: C, 78.06; H, 8.03; N, 8.81.

Hydrolysis/deuteronation experiments: D_2_O (*ca.* 100 μL) was added to a sample of **9b** (5 mg) in an aromatic solvent. Subsequent sonication gave a colourless solution and a fine white precipitate, presumably Mg(OD)_2_ D_2_O. Analysis by ^1^H (in deuterated benzene) and ^2^H (in benzene) NMR spectroscopy only showed resonances for 4-deuteropyridine and ^Dep^nacnacD (N*D* resonance in ^2^H-NMR spectrum: *δ* 12.1 ppm) as organic products. NMR resonances for 4-deutero-pyridine: ^1^H-NMR (400.1 MHz, C_6_D_6_) *δ* = 6.68–6.72 (m, vd, *J* = 4.5 Hz, 2H, 3-H), 8.49-8.53 (m, vd, *J* = 5.4 Hz, 2H, 2-H); ^2^H-NMR (76.7 MHz, C_6_H_6_) *δ* = 7.00 (br, vtr, 4-D). In a J.Young NMR tube, **9b** (6 mg) was treated with DMSO-*d*_6_ (0.5 mL). After shaking, the mixture dissolved, was analysed by ^1^H and ^2^HNMR spectroscopy, and showed the formation of 4-deuteropyridine and a highly symmetrical β-diketiminate product, likely [(^Dep^nacnac)Mg(OS{CD_3_}CD_2_)]. NMR resonances for 4-deutero-pyridine: ^1^H-NMR (499.9 MHz, DMSO-*d*_6_) *δ* = 7.37-7.40 (m, vd, *J* = 5.0 Hz, 2H, 3-H), 8.56-8.59 (m, vd, *J* = 5.6 Hz, 2H, 2-H); ^2^H-NMR (76.7 MHz, DMSO-*d*_6_) *δ* = 7.77 (br, 4-D). NMR resonances for [(^Dep^nacnac)Mg{OS(CD_3_)CD_2_}]: *δ* = 1.14 (t, *J*_HH_ = 7.5 Hz, 12H; CH_2_C*H*_3_), 1.45 (s, 6H; NCC*H*_3_), 2.48 (dq, *J*_HH_ = 15.0, 7.5 Hz, 4H; C*H*_2_CH_3_), 2.64 (dq, *J*_HH_ = 15.0, 7.5 Hz, 4H; C*H*_2_CH_3_), 4.56 (s, 1H; γ-C*H*), 6.92-7.02 (m, 6H; Ar-*H*).

### 4.4. X-ray Crystallographic Details

Suitable crystals were mounted in paratone oil and were measured using either a Rigaku FR-X Ultrahigh brilliance Microfocus RA generator/confocal optics with XtaLAB P200 diffractometer (Mo Kα radiation) or a Rigaku MM-007HF High Brilliance RA generator/confocal optics with XtaLAB P200 diffractometer (Cu Kα radiation). Data for all compounds analysed were collected using CrystalClear. [28] Data were processed (including correction for Lorentz, polarization and absorption) using either CrystalClear [28] or CrysAlisPro.[29] Structures were solved by a dual-space method (SHELXT-2018/2) [30] and refined by full-matrix least-squares against *F*^2^ using SHELXL-2018/3. [31] All non-hydrogen atoms were refined anisotropically except in selected cases as described below. Hydrogen atoms were placed in calculated positions (riding model). For solvates of **9a**, severely disordered solvent of crystallisation (benzene) was removed using the Platon/SQUEEZE routine [32] to estimate the solvent content and present images in the supporting information. All calculations were performed using CrystalStructure. [33] Details on individual crystal structure determinations and refinements are given below. Further experimental and refinement details are given in the CIF-files. CCDC 2119757-2119759 contains the supplementary crystallographic data for this paper. These data can be obtained free of charge via https://www.ccdc.cam.ac.uk/structures/.

Molecular structures of **9a**. Several datasets of benzene solvates of **9a** were collected of which information on two are briefly presented. Due to the poor overall diffraction and refinement data, only limited data are given here, and images are presented in the supporting information. In both cases, the Platon/SQUEEZE routine was used to estimate the disordered solvent content.

[{(^Dip^nacnac)Mg(4-C_5_H_4_N)}_6_]·10 C_6_H_6_, **9a**·10 C_6_H_6_. Crystallised with a sixth of the molecule in the asymmetric unit (Figure S1). C_264_H_330_Mg_6_N_18_, *M* = 3901.30, *T* = 123(2) K, Cu Kα, *Cubic*, *Ia*-3*d*, *a* = 46.1277(2) Å, *b* = 46.1277(2) Å, *c* = 46.1277(2) Å, *V* = 98148.9(13) Å^3^, *Z* = 16, Reflections collected: 339735, Independent reflections: 4740 [*R*_int_ = 0.0934]; *R*_1_ [*I* > 2σ(*I*)] ca. 15% (after use of SQUEEZE).

[{(^Dip^nacnac)Mg(4-C_5_H_4_N)}_6_]·16 C_6_H_6_, **9a**·16 C_6_H_6_. Crystallised with half of the molecule in the asymmetric unit (Figure S2). C_306_H_366_Mg_6_N_18_, *M* = 4442.01, *T* = 173(2) K, Mo Kα, *Triclinic*, *P*-1, *a* = 14.2254(3) Å, *b* = 21.0010(5) Å, *c* = 22.3351(8) Å, *α* = 79.966(3)°, *β* = 83.584(2)°, *γ* = 83.702(2)°, *V* = 6501.5(3) Å^3^, *Z* = 1, Reflections collected: 204082, Independent reflections: 29318 [*R*_int_ = 0.0944]; *R*_1_ [*I* > 2σ(*I*)] ca. 16.6% (after use of SQUEEZE).

Molecular structures of **9b**. Information on two similar datasets of **9b**·C_6_H_6_ are given, both contain one sixth of the molecule in the asymmetric unit. The first one was used in the discussion and is shown in Figure 3. In each case, one ethyl group is disordered and was modelled with two positions for the methyl group and their positions (73:27% or 71:29% parts) freely refined anisotropically using geometry restraints (DFIX).

[{(^Dep^nacnac)Mg(4-C_5_H_4_N)}_6_]·C_6_H_6_, **9b**·C_6_H_6_. CCDC 2119757, C_186_H_228_Mg_6_N_18_, *M* = 2861.80, *T* = 125(2) K, Cu Kα, *Trigonal*, *R*-3, *a* = 33.5457(4) Å, *b* = 33.5457(4) Å, *c* = 13.08330(16) Å, *V* = 12750.3(3) Å^3^, *Z* = 3, ρ = 1.118 Mg/m^3^, *F*(000) = 4626, theta range: 2.634 to 75.779°, indices −41 ≤ *h* ≤ 41, −41 ≤ *k* ≤ 41, −16 ≤ *l* ≤ 16, Reflections collected: 50493, Independent reflections: 5822 [*R*_int_ = 0.0370], Completeness to theta (67.684°): 99.9%, Goof: 1.054, Final *R* indices [*I* > 2σ(*I*)]: *R*_1_ = 0.0449, *wR*_2_ = 0.1392, *R* indices (all data): *R*_1_ = 0.0509, *wR*_2_ = 0.1457, Largest diff. peak and hole: 0.24 and −0.24 e∙Å^−3^.

[{(^Dep^nacnac)Mg(4-C_5_H_4_N)}_6_]·C_6_H_6_, **9b**·C_6_H_6_. CCDC 2119758, C_186_H_228_Mg_6_N_18_, *M* = 2861.80, *T* = 173(2) K, Mo Kα, *Trigonal*, *R*-3, *a* = 33.599(3) Å, *b* = 33.599(3) Å, *c* = 13.0701(10) Å, *V* = 12778.0(19) Å^3^, *Z* = 3, ρ = 1.116 Mg/m^3^, *F*(000) = 4626, theta range: 1.708 to 25.393°, indices −40 ≤ *h* ≤ 40, −40 ≤ *k* ≤ 40, −15 ≤ *l* ≤ 15, Reflections collected: 39441, Independent reflections: 5225 [*R*_int_ = 0.0481], Completeness to theta (25.242°): 99.9%, Goof: 1.378, Final *R* indices [*I* > 2σ(*I*)]: *R*_1_ = 0.0527, *wR*_2_ = 0.1582, *R* indices (all data): *R*_1_ = 0.0758, *wR*_2_ = 0.1820, Largest diff. peak and hole: 0.30 and −0.26 e∙Å^−3^.

Molecular structure of **10**·0.5 C_5_H_12_. A full molecule is present in the asymmetric unit. Two isopropyl groups are disordered and were modelled with two positions for each atom in the group (55:45% and 42:58% parts) and were freely refined anisotropically using geometry restraints (DFIX). The solvent molecule is disordered, was modelled using geometry restraints (DFIX, DANG), and was refined isotropically.

[(^Dip^nacnac)Mg(DMAP)(NMe_2_)]·0.5 C_5_H_12_, **10**·0.5 C_5_H_12_. CCDC 2119759, C_40.5_H_63_MgN_5_, *M* = 644.28, *T* = 173(2) K, Mo Kα, *Tetragonal*, *I*4_1_/*a*, *a* = 22.2072(6) Å, *b* = 22.2072(6) Å, *c* = 34.0161(9) Å, *V* = 16775.4(8) Å^3^, *Z* = 16, ρ = 1.020 Mg/m^3^, *F*(000) = 5648, theta range: 1.765 to 25.348°, indices −24 ≤ *h* ≤ 26, −26 ≤ *k* ≤ 25, −40 ≤ *l* ≤ 40, Reflections collected: 44417, Independent reflections: 7648 [*R*_int_ = 0.0138], Completeness to theta (25.242°): 99.6%, Goof: 1.019, Final *R* indices [*I* > 2σ(*I*)]: *R*_1_ = 0.0442, *wR*_2_ = 0.1303, *R* indices (all data): *R*_1_ = 0.0485, *wR*_2_ = 0.1361, Largest diff. peak and hole: 0.29 and −0.23 e∙Å^−3^.

## Data Availability

X-ray crystallographic data are available via the CCDC; please see the X-ray Section 4.4.

## Supplementary Materials

The following are available online, Figure S1: Molecular structure of [{(^Dip^nacnac)Mg(4-C_5_H_4_N)}_6_]·10 C_6_H_6_, **9a**·10 C_6_H_6_; Figure S2: Molecular structure of [{(^Dip^nacnac)Mg(4-C_5_H_4_N)}_6_]·16 C_6_H_6_, **9a**·16 C_6_H_6_. Solvent molecules and hydrogen atoms omitted.
