# Supplementary material for: Synthesis of a Hexameric Magnesium 4-pyridyl Complex with Cyclohexane-like Ring Structure via Reductive C-N Activation
Source: Molecules. 2021 Nov 28;26(23):7214. doi: 10.3390/molecules26237214 (PMC8658945; doi:10.3390/molecules26237214)
Supplement: Supplementary file 1 [file molecules-26-07214-s001.zip › molecules-1471143-supplementary.pdf]

Supporting Information

for

Synthesis of a hexameric magnesium 4-pyridyl complex with cyclohexane-like ring structure via reductive C-N activation

Samuel R. Lawrence,[1] Matthew de Vere-Tucker,[1] Alexandra M. Z. Slawin,[1] Andreas Stasch[1,*]

1 EaStCHEM School of Chemistry, University of St Andrews, North Haugh, St Andrews, KY16 9ST, United Kingdom. E-mail: as411@st-andrews.ac.uk.

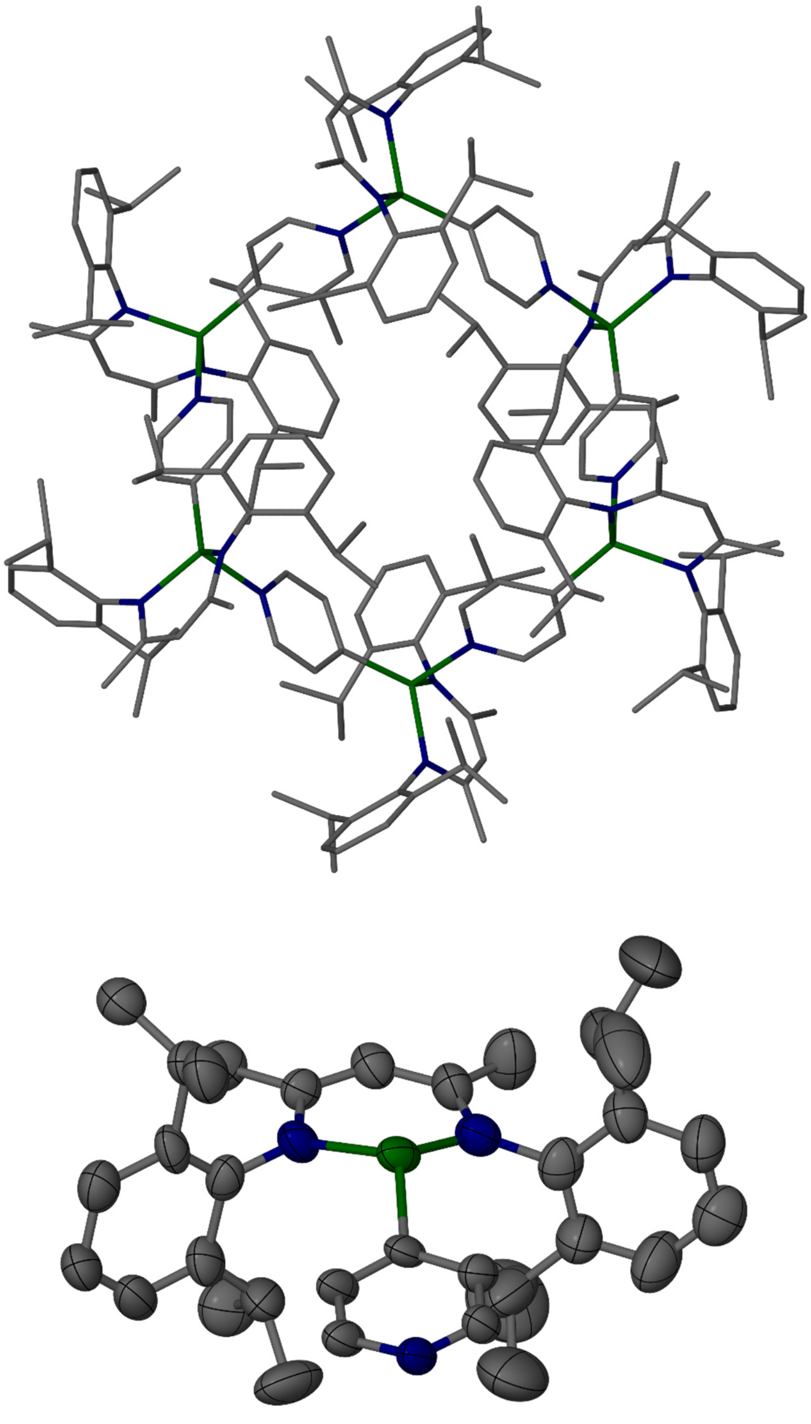

Figure S1. Molecular structure of [{(^{Dip}nacnac)Mg(4-C_5H_4N)}$_6$]·10 C_6H_6, **9a**·10 C_6H_6. Solvent molecules and hydrogen atoms omitted. Colour code: Mg: green, N: blue, C: grey. Top: Stick diagram of overall structure; Bottom: asymmetric unit, as thermal ellipsoids (30%). Due to the poor overall crystal and refinement quality, no bond distances are given. Cubic, *Ia*-3*d*, final *R* value *ca.* 15.0% after use of SQUEEZE (Per unit cell, 7123 electrons were removed in a total potential solvent accessible void volume of 30044 Å^3 (*ca.* 188 Å^3 per benzene molecule), which represents 30.6% of total cell volume).

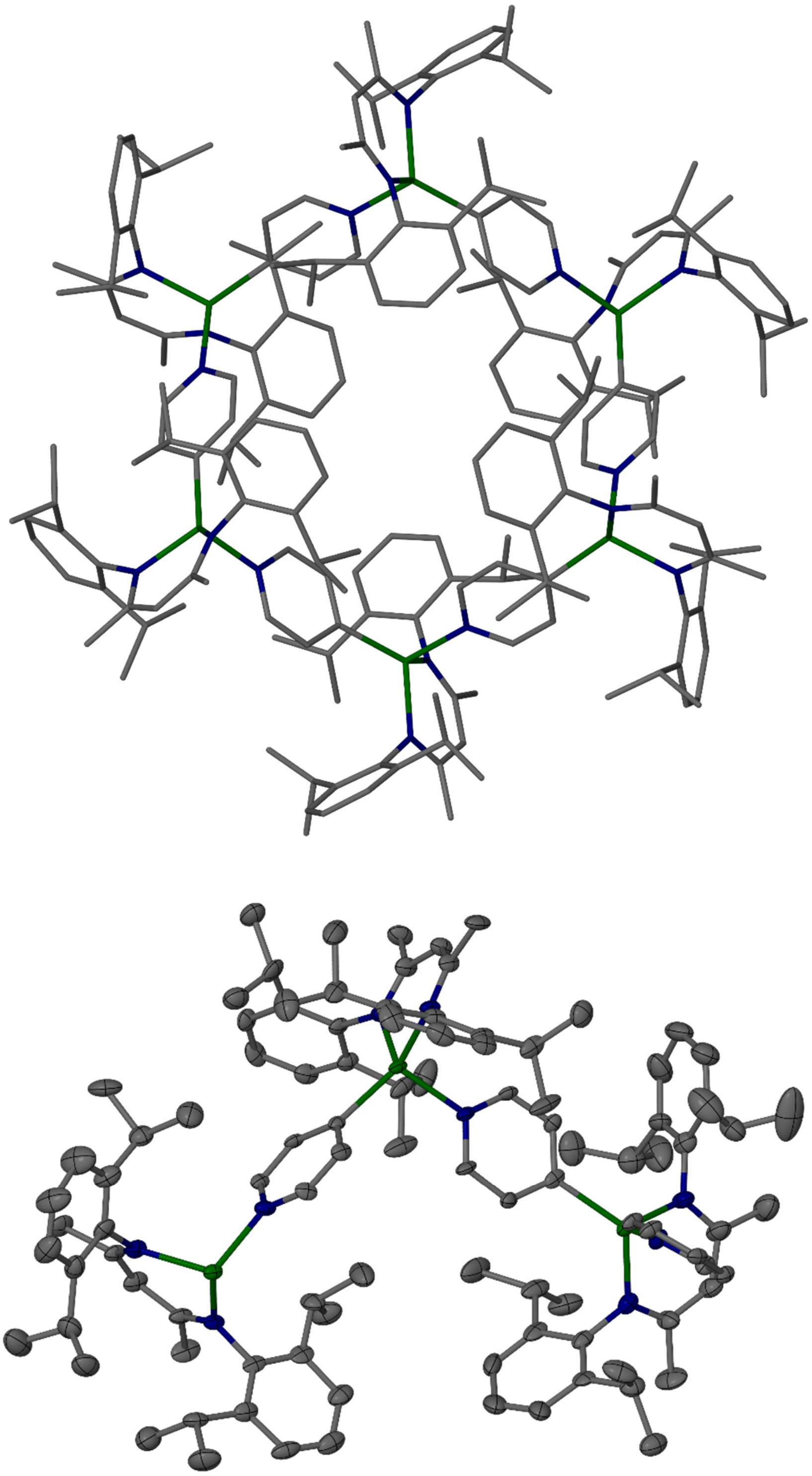

Figure S2. Molecular structure of $[\{(^{Dip}nacnac)Mg(4\text{-}C_5H_4N)\}_6]\cdot16\ C_6H_6$, **9a**$\cdot16\ C_6H_6$. Solvent molecules and hydrogen atoms omitted. Colour code: Mg: green, N: blue, C: grey. Top: Stick diagram of overall structure; Bottom: asymmetric unit, as thermal ellipsoids (30%). Due to the poor overall crystal and refinement quality, no bond distances are given. Triclinic, *P*-1, final *R* value *ca.* 16.6% after use of SQUEEZE (Per unit cell, 672 electrons were removed in a total potential solvent accessible void volume of 2378 Å^3 (*ca.* 149 Å^3 per benzene molecule), which represents 36.6% of total cell volume).
